# Torsion of an Abdominal-Wall Pedunculated Lipoma: A Rare Differential Diagnosis for Right Iliac Fossa Pain

**DOI:** 10.1155/2013/587380

**Published:** 2013-05-23

**Authors:** Daniel Lee John Bunker, Victor George Ilie, Tushar K. Halder

**Affiliations:** ^1^Department of Surgery, Royal Prince Alfred Hospital, Camperdown, Sydney, NSW 2050, Australia; ^2^Department of Surgery, Canterbury Hospital, Campsie, Sydney, NSW 2194, Australia

## Abstract

Pedunculated lipomas arising from the peritoneal wall are a rare finding during abdominal surgery. These benign tumours of mesenchymal origin can arise anywhere in the body and are usually asymptomatic. We present a case of a torted, pedunculated parietal wall lipoma in the right iliac fossa that gave rise to a clinical diagnosis of appendicitis. To our knowledge, such a case has never been reported in the literature previously. We suggest that torsion of a pedunculated parietal lipoma is a rare differential of acute abdominal pain.

## 1. Introduction

Torsion of intra-abdominal lipomas arising from the mesentery, omentum, and epiploic appendices has been reported as a cause of acute abdominal pain [1–5]. However, lipomas arising from the parietal peritoneum are a rare finding [6]. We present a case of a patient presenting with right iliac fossa pain clinically mimicking appendicitis which was found to be due to a torted, pedunculated parietal wall lipoma at laparoscopy. This is the first time such a case has been reported. The presentation, surgical finding, and management are discussed.

## 2. Case 

A 34-year-old female was referred to the local emergency department by her general practitioner following three days of increasing right iliac fossa pain. The pain was initially described as intermittent but became constant and exacerbated by movement, including coughing. Appetite was significantly decreased and she had two episodes of bloodless diarrhea. She had no fevers, upper respiratory tract symptoms, or urinary symptoms. Her last menstrual period was two days prior to the presentation. Her past medical history included anemia, hepatitis B carrier state, and uncomplicated delivery of a healthy infant two months previously. She was on no regular medications. 

On examination she was normotensive and afebrile. She was noted to have abdominal tenderness in the right iliac fossa with localized rebound and guarding. The rest of her examination was unremarkable. Urinalysis was positive for blood. Routine pathology showed a normal white cell count of 6.5 × 10^7^/litre, C-reactive protein was not performed, and beta-human chorionic gonadotropin was negative. 

**Figure 1:**
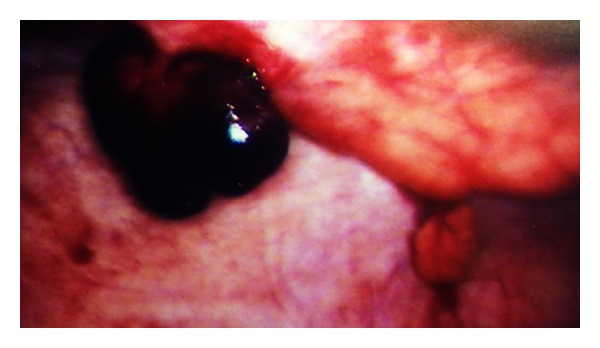
Intraoperative image showing the symptomatic lipoma. A second uncomplicated parietal wall lipoma is also aparent; it was also removed at the time of surgery.

The differential diagnoses in the emergency department were appendicitis, ovarian pathology, or renal colic. She was seen by the surgical registrar who felt her presentation was consistent with acute appendicitis and she was fasted, commenced on intravenous fluids and antibiotics, and prepared for theatre. She proceeded to a laparoscopic appendicectomy that evening. Upon entering the peritoneal cavity, blood was noted in the pelvis. The appendix was not grossly inflamed and the uterus, fallopian tubes, and ovaries were macroscopically normal. On the right lower quadrant of the anterior abdominal wall a torted pedunculated lipoma, with acute haemorrhage was noted (see Figure 1). A second untorted pedunculated lipoma was noted inferior-medially and both were resected laparoscopically. Laparoscopic appendicectomy was also performed. Antibiotics were ceased and the patient was discharged day one postoperatively, after an uneventful recovery. 

## 3. Discussion

Right iliac fossa pain is a classic sign of acute appendicitis, which remains the most common abdominal surgical emergency [7]. Differential diagnoses for right iliac fossa pain include mesenteric adenitis, diverticulitis, ureteric colic, Meckel's diverticulitis, Crohn's disease, leaking duodenal ulcer, biliary disease, and epiploic appendagitis. In women, endometriosis, pelvic inflammatory disease, salpingitis, and ovarian pathology also need to be excluded. As seen in this case, a torted pedunculated lipoma arising from the parietal peritoneum is another rare cause of right iliac fossa pain, which can mimic appendicitis clinically.

Lipomas are the most common neoplasm of mesenchymal origin and can arise anywhere in the body [6, 8]. They can arise from either deep or superficial structures and be single or multiple (lipomatosis). Most arise between 40 and 60 years of age and are slow growing, benign tumours [6]. They have a predilection for the trunk and are the most common tumour of the abdominal wall [9]. Those in the gastrointestinal tract usually arise from the submucosa or serosa. Gastrointestinal lipomas greater than 20 mm may produce abdominal pain, intussusception, altered bowel habit, or gastrointestinal blood loss [10].

Macroscopically, lipomas are soft, yellow or tan coloured mobile structures which are generally well defined from surrounding tissues. Histologically, they are composed of well-defined adipose tissue with a fibrous capsule. Lipomas can be detected clinically when superficial or radiologically when deep. Clinically, they appear as a soft and mobile mass. On ultrasound they appear as iso- to hyperechoic texture (when compared to the adjacent muscles), surrounded by a thin, echogenic capsule [11]. Deep lipomas can be reliably diagnosed on CT, where they appear as a well-circumscribed submucosal mass with uniform fat attenuation [12]. When treatment of lipoma is warranted, complete excision is advised. The acceptable recurrence rate is less than 5% [6].

To our knowledge, no other cases of a torted pedunculated abdominal wall lipoma mimicking acute appendicitis have been reported. A similar case was reported by Barut et al. [6] in a patient presenting with abdominal pain, nausea, and constipation who was found to have a pedunculated lipoma of the parietal peritoneum at laparotomy. Primary parietal tumours such as seen in this case are extremely rare [6]. Torsion of intra-abdominal lipomas arising from mesentery, omentum, and epiploic appendices have been reported as causes of abdominal pain [1–5]. 

While appendicitis remains a clinical diagnosis, early diagnostic laparoscopy has been advocated in acute abdominal pain as it has been shown to increase diagnostic accuracy as well as provide an avenue for surgical intervention [13]. In particular, it has been shown to decrease the rate of negative appendicectomy in young women, such as our patient [14]. In a negative diagnostic laparoscopy, there is merit in removing the normal looking appendix, as almost a third of macroscopically normal appendices will show inflammation on histopathology [15]. In our case, it was felt prudent to remove the macroscopically normal appendix and untorted pedunculated parietal peritoneal lipoma along with the torted lipoma, in an attempt to avoid a future operation.

While parietal peritoneal lipomas are rare, they remain a differential diagnosis for acute abdominal pain. Pain is usually associated with torsion of the lipoma around its pedicle. In this case, the location of the torted lipoma gave rise to a clinical diagnosis of appendicitis. 

While CT is useful for diagnosing acute appendicitis, most intra-abdominal lipomas are found at time of surgery. The exploratory laparoscopy is the best diagnostic method for a torted pedunculated lipoma. Intraoperatively, the surgeon should carefully examine the parietal peritoneal wall if the appendix is macroscopically normal, in order to exclude this rare differential diagnosis of acute appendicitis. Symptomatic intra-abdominal lipomas are best treated by total excision.

## References

[1] Farmlett EJ, Fishman EK, Jones B, Siegelman SS (1985). Case report. Torsion of lipoma of appendix epiploica: CT evaluation. *Journal of Computer Assisted Tomography*.

[2] Wolko JD, Rosenfeld DL, Lazar MJ, Underberg-Davis SJ (2003). Torsion of a giant mesenteric lipoma. *Pediatric Radiology*.

[3] Beattie GC, Irwin ST (2005). Torsion of an omental lipoma presenting as an emergency. *International Journal of Clinical Practice*.

[4] Macari M, Laks S, Hajdu C, Babb J (2008). Caecal epiploic appendagitis: an unlikely occurrence. *Clinical Radiology*.

[5] Aunan E, Naess A (1988). Torsion of intra-abdominal lipoma—a rare cause of recurrent or acute abdominal pain. Case report. *Acta Chirurgica Scandinavica*.

[6] Barut I, Tarhan OR, Ciris M, Tasliyar E (2006). Lipoma of the parietal peritoneum: an unusal cause of abdominal pain. *Annals of Saudi Medicine*.

[7] Birnbaum BA, Wilson SR (2000). Appendicitis at the millennium. *Radiology*.

[8] Weiss SW, Goldblum JR (2007). *Soft Tissue Tumous*.

[9] Gokhale S (2007). High resolution ultrasonography of the anterior abdominal wall. *Indian Journal of Radiology and Imaging*.

[10] Taylor AJ, Stewart ET, Dodds WJ (1990). Gastrointestinal lipomas: a radiologic and pathologic review. *American Journal of Roentgenology*.

[11] Truong S, Pfingsten FP, Dreuw B, Schumpelick V (1993). The value of ultrasound in the diagnosis of clinical doubtful findings of the abdominal wall and the inguinal region. *Chirurg*.

[12] Park SH, Han JK, Kim TK (1999). Unusual gastric tumors: radiologic-pathologic correlation. *Radiographics*.

[13] Golash V, Willson PD (2005). Early laparoscopy as a routine procedure in the management of acute abdominal pain: a review of 1,320 patients. *Surgical Endoscopy and Other Interventional Techniques*.

[14] Garbarino S, Shimi SM (2009). Routine diagnostic laparoscopy reduces the rate of unnecessary appendicectomies in young women. *Surgical Endoscopy and Other Interventional Techniques*.

[15] Phillips AW, Jones AE, Sargen K (2009). Should the macroscopically normal appendix be removed during laparoscopy for acute right iliac fossa pain when no other explanatory pathology is found?. *Surgical Laparoscopy, Endoscopy and Percutaneous Techniques*.

